# Varieties of Alexia From Fusiform, Posterior Inferior Temporal and Posterior Occipital Gyrus Lesions

**DOI:** 10.1155/2004/305194

**Published:** 2004-06-09

**Authors:** Yasuhisa Sakurai

**Affiliations:** Department of NeurologyMitsui Memorial Hospital1 Kanda-Izumi-choChiyoda-kuTokyo101-8643Japan

## Abstract

Reading impairments of three alexia patients, two pure alexia and one alexia with agraphia, due to different lesions were examined quantitatively, using Kanji (Japanese morphogram) words, Kana (Japanese phonetic writing) words and Kana nonwords. Kana nonword reading was impaired in all three patients, suggesting that widespread areas in the affected occipital and occipitotemporal cortices were recruited in reading Kana characters (corresponding to European syllables). In addition, the findings in patient 1 (pure alexia for Kanji and Kana from a fusiform and lateral occipital gyri lesion) and patient 2 (pure alexia for Kana from a posterior occipital gyri lesion) suggested that pure alexia could be divided into two types, i.e. ventromedial type in which whole-word reading, together with letter identification, is primarily impaired because of a disconnection of word-form images from early visual analysis, and posterior type in which letter identification is cardinally impaired. Another type of alexia, alexia with agraphia for Kanji from a posterior inferior temporal cortex lesion (patient 3), results from deficient whole-word images of words per se, and thus should be designated “orthographic alexia with agraphia”. To account for these impairments, a weighted dual-route hypothesis for reading is suggested.

## 1. Introduction

Pure alexia (alexia without agraphia) is defined as the inability to read printed material while writing is preserved [10]. Dejerine suggested that pure alexia is induced by any lesion that interrupts visual input to the angular gyrus, in which visual images of words and letters are stored [28,29]. Geschwind [40] stressed the importance of the splenium lesion in pure alexia. He claimed that damage to the splenium prevents the interhemispheric transmission of visual information in written language. However, subsequent lesion studies showed that pure alexia can occur without a splenium lesion [25,42]. In fact, this point had already been predicted by Dejerine. Greenblatt [41] classified this nonclassical type of alexia [51] into occipital alexia and subangular alexia, claiming that both types of alexia can be accounted for by the interruption of association fibers to the angular gyrus. Presently, the anatomic substrate of pure alexia is considered to be in the paraventricular white matter of the left occipital lobe [25], the lingual and fusiform gyri including the white matter [47], or the ventral occipitotemporal area [12]. Conversely, converging findings from neuroimaging studies have revealed that the ventral occipitotemporal or posterior inferior temporal cortices (Brodmann Area [BA] 37) are essential in reading [5,15,18,33,57,62,95].

Letter-by-letter reading observed in pure alexia has received attention from cognitive psychologists [8,64, 93,103]. The patients cannot read regular words (that obey spelling-sound rules) or irregular words and depend on letter-by-letter reading [36]. Therefore, longer words are read far more slowly than shorter words (word-length effect). Kinesthetic reading, i.e. tracing letters with the fingers, is a strategy for letter-by-letter readers to compensate for reading difficulty [10]. Letter-by-letter readers are impaired at letter identification [4,34,93], but some of them can make lexical decisions and semantic categorization, even though they cannot explicitly identify the stimulus [14,90,102]. Moreover, some patients show a frequency effect (high-

ISSN 0953-4180/04/$17.00 © 2004 – IOS Press and the authors. All rights reserved frequency words are recognized better or faster than low-frequency words) and imageability effect (imageable words are recognized better or faster than non-imageable words), which is more marked in longer words [8,22]. Theoretically, letter-by-letter reading was suggested to be derived from impaired ability to process letters by whole word recognition units, and thus was called word-form dyslexia [103]. According to this view, the word-form system is responsible for segmenting letter strings into recognizable units, such as letters, syllables, morphemes and words. Another suggestion was that letter-by-letter readers are not able to gain access to the orthographic lexicon from print [64].

The Japanese language has two distinct writing systems; i.e. Kanji (ideograms or morphograms, originally adopted from Chinese characters) and Kana (Japanese phonetic writing or syllabograms, originally taken from Kanji characters). Kana are further divided into Hira-kana (cursive Kana, examples here are all this form) and Kata-kana (square Kana that are used primarily for representing loan words). Kanji are graphically complicated and have meaning as well as two ways of being read: the *on*-reading that conveys the phonetic value; and *kun*-reading that conveys the meaning (e.g. 時 [ji] by *on*-reading and [toki] by *kun*-reading, meaning "time"). Kanji words consist of more than two Kanji or are accompanied by Kana suffixes, and usually have one reading attached to the meaning (e.g. 時計 [tokei], clock; 計る [hakaru], measure). Therefore, reading of Kanji words requires whole-word recognition as well as individual character identification. In contrast, Kana are graphically simple and have one definite phonetic value and no intrinsic meaning (e.g. と [to]). Kana words consist of one or more Kana and always have only one phonetic reading (e.g. とけい [tokei], clock). Therefore, reading of Kana words inevitably involves sequential letter-by-letter or character-by-character reading, although once the words become familiar, they can be read letter-by-letter or as whole words (discussed later). Due to the use of these dual systems, alexia in Japan can present as dissociative reading disturbances between Kanji and Kana [49]. For example, Kanji reading is selectively impaired in alexia with agraphia [49,53,85] or pure agraphia [92] caused by a lesion in the fusiform and inferior temporal gyri (posterior inferior temporal area; Area 37), whereas Kana reading is believed to be more disturbed in alexia with agraphia caused by a lesion in the angular gyrus [49,52,104].

Alexia with agraphia for Kanji is defined as reading and writing impairment that is predominantly disturbed for Kanji. In a literature review, Kanji reading varied from 8 to 91% correct, whereas Kana reading was 75% correct or greater [85]. In some patients, alexia improved to a normal range and agraphia alone remained. In this sense, it is called pure agraphia for Kanji [92]. Alexia with agraphia for Kanji from a posterior inferior temporal cortex lesion is said to parallel lexical agraphia in European countries [92], because lexical agraphia is defined as agraphia for irregular words [6], and Kanji characters are comparable to orthographically irregular or ambiguous words [92] (this view is open to question, see Discussion). It remains unknown whether alexia with agraphia for Kanji [49,53,85], pure agraphia for Kanji [92] and lexical agraphia [6] are equivalent. Based on the fact that they all arise from Area 37 insult, it is suggested that they share the same underlying mechanism. Kana reading is not or less disturbed in this type of alexia. However, some patients read Kana more poorly than Kanji with a similar lesion [51,61,91]. It was argued that when Kana reading impairment is more pronounced, the lesion extended from Area 37 posteriorly to the lateral occipital gyri [85].

Conversely, in alexia with agraphia from an angular gyrus lesion, a patient with an infarction limited to the angular gyrus did not show alexia [86]. We claimed that alexic symptoms in "angular" alexia with agraphia may be the result of lesion extension to the posterior occipital gyri [87], damage to which causes pure alexia selectively impaired for Kana [77] (described below). Kleist [54] first attributed the alexic symptom in alexia with agraphia to lesion extension from the angular gyrus to the middle occipital gyrus, immediately posterior to the angular gyrus.

Letter-by-letter reading in pure alexia has rarely been described in Japan. This is probably because there is no such a compensatory strategy available as spelling the word aloud in the Japanese language. However, reading of Kana, which essentially involves the sequential letter-by-letter process (described above), becomes slow and laborious. Letter-by-letter reading, kinesthetic facilitation and inability to read even what the patient just wrote have been regarded as hallmarks of pure alexia. In addition, pure alexia in Japan is known to impair Kanji reading and Kana reading to varying degrees [52,94,98]. This variety of reading between Kanji and Kana is observed in both classical type pure alexia (with a splenium lesion) and non-classical type pure alexia (without a splenium lesion) [52]. In some patients with non-classical type pure alexia, Kana reading is more disturbed than Kanji reading [51,99].

However, reading impairment limited to Kana has not been reported. We recently reported patients with pure alexia for both Kanji and Kana [87] and pure alexia for Kana only [77]. Pure alexia for Kanji and Kana showed damage to the fusiform gyrus and lateral occipital gyri, whereas pure alexia for Kana showed damage to the posterior occipital areas mainly involving the inferior occipital/fusiform gyri (Area 18/19). Since the patient with pure alexia from the fusiform gyrus lesion showed slight agraphia for Kanji due to impaired character recall and a patient with alexia with agraphia from a more lateral fusiform/inferior temporal gyri lesion showed similar but severer agraphia, we hypothesized that pure alexia for Kanji and Kana occurred when the fusiform gyrus was disconnected from the posterior inferior temporal area (lateral fusiform/inferior temporal gyri), where visual images of words were thought to be stored [87]. Pure alexia for Kana from a posterior occipital cortex lesion cannot be explained by this view. It was suggested that visual images of Kana are widely distributed in the ventral and lateral occipital gyri [77]. It was also suggested that there is functional specialization in the left occipital and occipitotemporal areas for reading Kanji and Kana; the posterior inferior temporal area for Kanji and the posterior occipital gyri for Kana.

In the present study, the reading ability of three patients with alexia with/without agraphia was examined further using Kanji words, Kana words and Kana nonwords. The study investigated the correlation of lesions in these patients with the activated sites shown in our previous PET studies [79,80,82,83] and proposed an anatomically-based model of reading that accounts for pure alexia from different lesions and alexia with agraphia due to a posterior inferior temporal gyri lesion.

## 2. Methods

### 2.1. Subjects

Three patients with alexia underwent a reading test. The patient profiles are as follows. Patient 1 (pure alexia for Kanji and Kana) [87]: a 73-year-old, right-handed man had a cerebral infarction in the left lingual gyrus in May 1995, although he was not aware of any visual field defect. In April 1996 he sustained a second stroke involving the left fusiform and lateral occipital gyri, after which he could no longer read any Kana and certain Kanji symbols. He read slowly and with difficulty even words that he had just written. A neurological examination showed right upper homonymous quadrantanopsia. Quantitative evaluation with the basic 100 Kanji (one-character words) and the corresponding Kana (Kana transcription of Kanji words) [85] two months after the second stroke revealed that he read 98% of Kanji and 97% of Kana correctly, although he spent more than five times longer in reading compared with normal controls.

Patient 2 (pure alexia for Kana) [77]: a 77-year-old, right-handed man became aware of a narrowed visual field and later noticed difficulty in reading Kana after a cerebral hemorrhage under the left posterior occipital cortices, mainly affecting the inferior occipital/fusiform gyri in March 1998. He even had difficulty reading sentences written by himself. A neurological examination showed right lower homonymous quadrantanopsia. He read Kana words character by character, and spent more time reading five-character Kana words than three-character Kana words (word length effect). Quantitative evaluation with the 100 Kanji and Kana described above two months after onset revealed that he read 99% of Kanji and 83% of Kana correctly, requiring 15 times longer than normal for Kana reading. He also showed a minimal deficit in discriminating between circles and ovals or between squares and rectangles of a similar size.

Patient 3 (alexia with agraphia for Kanji) [85,87]: a 71-year-old right-handed man suddenly realized that he could not remember the names of objects and could not read newspapers or write anything but his own name, following a cerebral hemorrhage under the left lateral fusiform and posterior inferior temporal gyri in June 1991. His visual field was intact. A neuropsychological examination showed alexia with agraphia preferentially disturbed for Kanji along with severe anomia. Quantitative evaluation with 100 Kanji and Kana three months after onset revealed that he read 20% of Kanji and 75% of Kana correctly, and correctly wrote from dictation 4% of Kanji and 59% of Kana. Alexia with agraphia and anomia persisted at re-evaluation five years later. Fig. 1 illustrates their lesions drawn on coronal images.

As a control subject for the visual field defect, patient 4 underwent the same test. This 76-year-old, right-handed man suffered a first attack of cerebral infarction in the left medial occipital gyri in December 2000, and a second infarction in the small area of the right medial occipital gyri in July 2001. The Goldmann perimetry and Tokyo Medical College Color Vision Test revealed that he had right upper homonymous quadrantanopsia and cerebral achromatopsia, but he did not show alexia or agraphia.

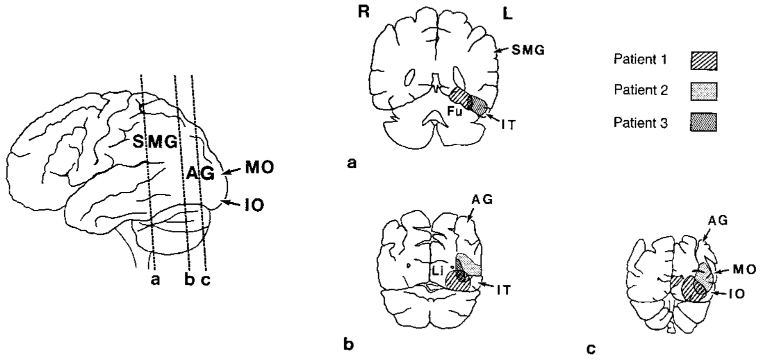

Fig. 1. Lesions of three alexia patients drawn on coronal images through the posterior inferior temporal cortex (a), angular gyrus (b) and lateral occipital gyri (c). The brain images were drawn from the atlas of Damasio [26]. Nomenclature is based on Talairach and Tournoux [96]. Patient 1: pure alexia (common type) from a lesion of the fusiform and lateral occipital gyri, Patient 2: pure alexia (posterior type) from a posterior occipital gyri lesion, Patient 3: alexia with agraphia for Kanji (orthographic alexia with agraphia) from a lesion of the lateral fusiform and inferior temporal gyri. Abbreviations: SMG, Supramarginal gyrus; AG, Angular gyrus; MO, Middle occipital gyrus; IO, Inferior occipital gyrus; Fu, Fusiform gyrus; IT, Inferior temporal gyrus; Li, Lingual gyrus.

### *2.2. Materials*

A reading test was provided, consisting of 100 three-character Hira-kana words (e.g. きそく [kisoku], rule), the corresponding two character Kanji words (e.g. 規則 [kisoku], rule), and 100 three-character Hira-kana nonwords (e.g. きひせ [kihise]). The Kana words were chosen from those with higher familiarity based on how often the subject had seen, heard or used the word (above 3.50, assessed with a five-point rating scale, range from 0.00 to 4.96) [55]. The Kana nonwords were made by combining Kana symbols that have no association with each other [2]. All Kana word and Kana nonword materials were included among those used in our PET reading studies (100 out of 240 word or nonword materials were selected) [79,80,82,83]. The mean familiarity value of Kanji words assessed with a seven-point rating scale (range from 1.031 to 6.812) in a recent research was 6.098 [3]. Patient 2 was further given the same test and an additional reading test of 100 three-character Kana word with low familiarity (range between 1.50 and 1.99 in the five-point rating scale, above described) at three years post-onset. Patient 3 was also given another set of Kana nonword reading test two years later.

### *2.3. Analysis*

Patients' oral responses and the time spent in reading were recorded. Errors were classified into non-response, visual, phonological, and semantic. In the English language based studies, visual errors have been tightly defined as responses in which there is at least a 50% overlap of the letters between the target and response [20]. These criteria, however, do not apply to Japanese script, in which a visual error occurs within one character. Errors were regarded as visual when they had the same component as the target character and thus resembled it visually, e.g. 都会 ([tokai], city) → 都合 ([tsugou], convenience). In Kanji, semantic and visual errors are sometimes difficult to distinguish, whereas in Kana, visual and phonological errors are sometimes difficult to distinguish. These errors were labeled as "semantic and/or visual" or "phonological and/or visual," according to the English classification [20] (for examples, see footnote of Table 2). In this regard, almost all phonological errors in Kana are visually similar to the target word (at least 50% overlap of the characters), e.g. しかい ([shikai], chairman) → しかく ([shikaku], square), where two of the three characters are the same. These were not classified into visual errors but phonological errors, because visual errors take place at a character level, as indicated above. When a character in a word was changed into another character visually similar to the character, it was classified as phonological and/or visual errors, e.g. にんき ([ninki], popularity) → こんき ([konki], patience). The present phonological errors probably correspond not only to phonemic paralexia errors but to visual and derivational errors in European languages.

A patient was regarded as impaired in reading, when the score was greater than 2 SD below the normal mean. If he had a visual field defect and read materials more than two times longer than the control patient with quadrantanopsia (patient 4), he still was regarded as impaired, irrespective of the score.

Table 1
Patient profiles and reading performance

| Patient | 1 | 2 | 3 | 4 | Controls ($N = 11$)[a] | |
|---|---|---|---|---|---|---|
| Age, Sex | 73M | 77M | 71M | 76M | mean score (SD) | mean time for reading |
| Symptom | Pure alexia (recovery phase) | Pure alexia | Alexia with agraphia | | | |
| Visual field defect | RU | RL | | RU | | |
| Reading impairment | Kanji and Kana | Kana > Kanji[b] | Kanji > Kana | | | |
| Lesion | Fu, Post O | Post O | Fu/Inf T | Med O | | |
| Time after onset (months) | 2 | 3 | 61–64 | 8 | | |
| Reading test of 100 two-character Kanji words, three-character Kana words and three-character Kana nonwords | | | | | | |
| Kanji word reading | 99 (5 min; 5/5)* | 96 (4 min; 1/2) | 52 (8 min; 2/15) | 100 (3 min) | 99 (0.9) | 1 min 19 sec |
| Kana word reading | 99 (4 min) | 82 (16 min; 40/45) | 84 (6 min) | 100 (3 min) | 98 (3.0) | 1 min 35 sec |
| Kana nonword reading | 81 (4 min) | 72 (10 min; 17/26)** | 45 (6 min)*** | 97 (4 min) | 95 (3.3) | 2 min 46 sec |

*Values are % correct (and time spent in the task; correct responses with kinesthetic reading). The fraction denotes the number of correct responses versus the total attempts at kinesthetic reading, e.g. 5/8 means that five were correct for all eight kinesthetic reading trials.
**In patient 2, Kana nonword reading was examined two weeks after the Kanji word reading and Kana word reading tests.
***In another set of Kana nonword reading test, patient 3 got the score of 67/100 in six minutes two years later.
[a]Normal controls: 10 men and 1 woman, ages 61 to 78, mean 68 years old, senior high school graduate normal volunteers who had no past history of neurological disorders [78].
[b]Kana word reading was more impaired than Kanji word reading.
Abbreviations. M, Man; RU, Right upper quandrantanopsia; RL, Right lower quadrantanopsia; Fu, Fusiform gyrus; Post O, Posterior occipital gyri; Inf T, Inferior temporal gyrus; Med O, Medial occipital gyri.

## 3. Results

Patient 1 (pure alexia for Kanji and Kana caused by a lesion in the medial fusiform and lateral occipital gyri) underwent the test two months after onset, when he became able to read newspapers. At this time, he read Kanji words more slowly than the control subject with quadrantanopsia (Table 1), though the reading time did not exceed two-fold the control patient's time. He read Kana nonwords significantly more poorly than Kana words ($p < 0.001$, by Fisher's exact method), but read Kana nonwords as fast as Kana words. All his reading errors were phonological ones (phonemic paralexia; Table 2); he misread one or two characters in a three-character Kana nonword, some of the errors apparently being due to visual similarity (phonological and/or visual error; e.g. あ [incorrect] for お [correct]).

Patient 2 (pure alexia or letter-by-letter reading caused by a lesion in the posterior occipital gyri) showed almost selective impairment of Kana reading ($p = 0.0026$, between Kanji words and Kana words by Fisher's exact method), although Kanji reading was slightly impaired. He read Kana nonwords more poorly than Kana words, but not significantly. Time for reading Kanji was within the normal range or slightly longer than that of the control patient. Most Kana reading errors were phonological and phonological and/or visual ones. In the re-examination with the additional reading test at three years post-onset, he still read Kana words and nonwords character by character. The scores were 99/100 for Kanji (time for reading, 3 min), 99/100 for Kana words (5 min), 97/100 for Kana words with low familiarity (5 min), 91/100 for Kana nonwords (5 min), suggesting a familiarity effect in reading (familiar words are read better than unfamiliar words).

Patient 3 (alexia with agraphia for Kanji caused by a lesion in the lateral fusiform and inferior temporal gyri) exhibited severe reading impairments of Kanji words with relative sparing of Kana word reading ($p < 0.0001$ by Fisher's exact method). However, he read Kana nonwords markedly more poorly than Kana words ($p < 0.0001$ by Fisher's exact method), although he read Kana nonwords as fast as Kana words. Most Kanji reading errors were non- or partial responses (impaired word recall) and there was only one semantic error. Conversely, most Kana reading errors were phonological and phonological and/or visual ones.

## 4. Discussion

First, how the Japanese writing system is related to European languages is briefly described. Phonologically, Kana symbols parallel European consonant-vowel syllables or mora in the sense that they develop one-to-one character-to-sound correspondence. Orthographically, Kana characters correspond to European letters. Kana character strings are regular, whether they are words or nonwords, since there is no pronunciation ambiguity in translating Kana characters to their cor-

Table 2
Types of reading errors in patients 1 to 3 in the reading test (values are number of error responses)

| Patient | 1 | 2 | 3 |
|---|---|---|---|
| Kanji word ($n = 100$) | | | |
| Non-response | | | 7 |
| Partial response* | 1 | 3 | 24 |
| *On-kun* confusion | | | 2 |
| Semantic | | | 1 |
| Semantic and/or visual | | 1 | |
| Unrelated | | | 14 |
| Total errors | 1 | 4 | 48 |
| Kana word ($n = 100$) | | | |
| Non-response | | | 1 |
| Partial response** | | 2 | 2 |
| Phonological | 1 | 10 | 10 |
| Phonological and/or visual | | 6 | 3 |
| Total errors | 1 | 18 | 16 |
| Kana nonword ($n = 100$) | | | |
| Partial response** | | 1 | 1 |
| Phonological | 15 | 16 | 41 |
| Phonological and/or visual | 4 | 11 | 13 |
| Total errors | 19 | 28 | 55 |

*Partial response: one symbol of a two-character Kanji word could not be read or was misread. *On-kun* confusion: a patient read a Kanji word irrespective of its meaning. As a result, a Kanji word was read in a way different from the original reading, e.g.見本([mihon], sample) → [mimoto], where 本 is read as [moto] (*kun*-reading; a type of reading of a Kanji symbol that conveys its meaning in the Japanese language) instead of [hon] (*on*-reading; another type of reading of a Kanji symbol that conveys its phonetic value according to the Chinese pronunciation). Semantic errors: those semantically associated with the target word, e.g. 今夜 ([konya], tonight) → 昨夜 ([sakuya], last night). Some semantic errors included changing a character into another character visually similar to a target character (semantic and/or visual), e.g. 月日 ([tsukihi], months and days) → 明日 ([asu], tomorrow). Unrelated response: the changing of a Kanji into another Kanji that has no visual or phonological similarity to the correct answer.

**Partial response: one or more Kana characters of a Kana word could not be read. Phonological response: one or more Kana characters of a Kana word were substituted for other Kana (phonemic paralexia). Some phonological errors included changing a character into another character visually similar to a target character (phonological and/or visual), e.g. けんい ([keni], authority)→ はんい ([hani], extent).

responding sounds. Thus, Kana words are comparable to regular words, and Kana nonwords are comparable to regular nonwords (pseudowords) [78]. Conversely, single Kanji characters correspond to mora too, but they are irregular, because there is nearly no character-to-sound correspondence. However, Kanji words, consisting of two or more Kanji characters, have some subtle regularities (or consistencies, according to the connectionist terminology) [39]. Therefore, strictly speaking, Kanji words are comparable to real words (regular and irregular words).

Next, the semiological features of the alexic patients are discussed. Then, anatomical substrates of alexia and a modified dual-route model for reading are proposed to show how these reading impairments are explained with this model.

### 4.1. Neuropsychological considerations

Kana nonword reading was impaired in all three patients, even when Kana word reading seemed to be preserved (patients 1 and 3). Single Kana character reading was also disturbed during the course in all patients [77,87]. These findings suggested that widespread areas in the fusiform (Areas 19 and 37), inferior temporal and lateral occipital gyri were recruited in reading Kana characters (not specific for Kana nonwords). It is also suggested that mere comparison between Kanji words and Kana words is not enough to demonstrate dissociation between two scripts, which has been overlooked in the previous studies.

Prominent Kana nonword reading impairment and deficient Kana character identification are also observed in phonological dyslexia [66,88], in which nonword reading is selectively impaired. In particular, patient 3 made a large number of nonword reading errors, which is attributable to reduced activation of phonological representations [66]. Thus, the present results suggest that pure alexia and alexia with agraphia should be differentiated from phonological dyslexia, and vice versa. The dyslexic symptom of the present patients is different from phonological dyslexia in that they read Kana nonwords as fast as Kana words (Table 1). In contrast, a patient with phonological dyslexia [88] spent more time reading Kana nonwords than Kana words. This difference in reaction time for Kana nonwords suggests that in the present patients, impaired character identification determined the rate of reading, whereas this was not the case in phonological dyslexia. Therefore, the present patients' difficulty reading nonwords was probably derived from impaired recognition of Kana symbols. When patient 3 was given another set of Kana nonword reading test two years later, the difference between Kana word and nonword reading was found to be smaller (see footnote of Table 1).

Impairment of character identification was not evident in Kana word reading, probably because the patients used lexical (orthographic, phonological or semantic) help or "lexical capture" [38,66] in reading. Lexical capture is a lexical analogy strategy to read unfamiliar words or nonwords with reference to lexical knowledge, orthographic, phonological or semantic [38]. Namely, even if they had difficulty identifying one character in a word, they could guess the character from the context. In fact, patient 1 reported that he used a semantic strategy while reading. Probably the same mechanism worked in patients 2 and 3, which was reflected in their performances in reading Kana words better than Kana nonwords.

The presence of pure alexia for Kanji and Kana (patient 1) and pure alexia for Kana (patient 2) suggests that pure alexia can be divided into two types; one for deficient whole-word recognition and letter or character identification due to a fusiform gyrus lesion (ventromedial type; Table 3); and the other for deficient letter or character identification solely from a posterior occipital gyri lesion (posterior type; Table 3). Patient 1 had both fusiform and posterior occipital cortex lesions, thus, it remains unclear which lesion affected his Kana character reading. Letter-by-letter reading may be more evident in the posterior occipital gyri lesion, as in patient 2, probably because this region is more specific for letter or character identification [77].

According to a dual-route model of reading [31], alexia can arise from a deficit in early visual analysis, parallel access to word forms, word forms themselves, or some combination of these loci. In the light of this view, patient 1 had damage to access to word forms, whereas patient 3 had damage to word forms themselves that are located in the posterior inferior temporal cortex [87]. The difference of deficits between "access to word forms" and "word forms themselves" is that in a word-form deficit severe agraphia due to impaired character recall coexists and kinesthetic reading is less effective (described in 4.3) whereas in an access deficit the patient can write a Kanji he cannot read [87].

Patient 2 had a deficit in character identification. Letter identification is thought to be included in visual analysis [31]. Thus, impaired Kana character reading in patient 2 can be attributed to damage to the visual analysis system. Conversely, relatively preserved Kanji word reading implies that whole-word reading, which is expected to be more involved in reading of graphically complex characters such as Kanji, was still possible in this patient. He had difficulty reading Kana words, suggesting that Kana word reading depends less on whole-word recognition than Kanji word reading. Deficient character identification in contrast to relatively preserved whole-word reading suggests that there is a little serial connection from letter/character identification to word form representations: a process that links to word forms (orthographic input lexicon) [31] is probably not letter identification but an earlier visual analysis consisting of line and contour detection of linguistic and nonlinguistic stimuli. Instead, letter/character identification directly links to grapheme-phoneme conversion, because reading a Kana character aloud inevitably involves letter/character identification and grapheme-phoneme conversion and it is difficult to evaluate separately these two processes only with a reading test. Therefore, we can safely state that patient 2 had damage to the early visual-phonological route. This view is supported by our PET studies (Fig. 2 [80, 81]) in which the inferior occipital/fusiform gyri (Area 18/19) were specifically activated in Kana word covert reading, and more extensive area (including the middle occipital gyrus) was activated in the Kana word reading aloud task. These gyri probably constitute a continuous but functionally distinct, visual (inferior occipital) to phonological (middle occipital), route.

A problem here is that a single Kana character reading was impaired in any type of alexia from different lesions (patients 1 to 3). This fact suggests that visual images of Kana characters are widely distributed in the ventral and lateral occipital gyri (described in the introduction) and are used for both phonology (grapheme-phoneme conversion) and orthography (whole-word recognition). Thus, in general letter/character identification should not be confined to visual analysis. Furthermore, the inferior occipital/fusiform gyri (Area 18/19) are more specified to identify Kana characters (patient 2) and constitute a functionally independent module that is separable from early visual analysis and links to grapheme-phoneme conversion.

### *4.2. Neural basis of reading*

We conducted PET reading studies using Kanji words, Kana words and Kana nonwords separately in different groups (Fig. 2) [79–83] and found that: (i) in addition to the posterior superior temporal gyrus, the basal occipital and occipitotemporal areas (Areas 18, 19 and 37) were activated by a conjunction [71] involving Kanji and Kana, suggesting that these areas are crucial for real word reading; (ii) activity was more pronounced in Area 37 in Kanji, which was suggested to be concerned with the orthography of Kanji, in contrast to greater activation in Areas 18 and 19 in Kana; (iii) specific activation for a conjunction involving Kana words and nonwords was located in the middle occipital gyrus and the deep perisylvian temporoparietal cortex (Areas 22/21 and 40/22), suggesting that these areas are engaged in phonological processing of Kana character sequences; and (iv) Kana nonwords activated more

Table 3
Types of alexia and lesion sites

| Alexia | Orthographic* | Visual-phonological* | Complications | References |
|---|---|---|---|---|
| Pure alexia: ventromedial type** | I (Fu)[a] | | rt upper quadrantanopsia | [100] |
| Pure alexia for Kanji and Kana (Common type; pt 1) | I (Fu)[a] | I (Fu/Inf O)[b] | rt upper quadrantanopsia or rt hemianopsia | [30,44,51,57,99,101,103] |
| Pure alexia for Kana: posterior type (Pure alexia for nonwords; pt 2) | | I (Fu/Inf O)[b] | rt lower quadrantanopsia | [8,17] |
| Alexia with agraphia for Kanji from PIT lesion (Orthographic alexia with agraphia; pt 3) | I (Fu/Inf T)[a] | | anomia | [24,49,53,74,92] |

*The orthographic pathway (ventral route) proceeds from the primary visual cortex to the inferior temporal gyrus (Area 37) via the fusiform gyrus (Area 37). The phonological pathway (dorsal route) proceeds from the primary visual cortex to the superior temporal gyrus and inferior supramarginal gyrus via the inferior and middle occipital gyri (Area 18/19) and deep perisylvian temporoparietal cortex (Areas 22/21 and 40/22).
**The ventromedial type of pure alexia refers to alexia in which whole-word reading is primarily impaired, and letter-by-letter reading is not so pronounced. The posterior type of pure alexia is identical with letter-by-letter reading in a strict sense, with preserved whole-word reading. Many cases of pure alexia have both lesions, as in pure alexia for Kanji and Kana.
[a]Area 37; [b]Area 18/19.
Abbreviations: I, Impaired; pt, patient; rt, right; PIT, Posterior inferior temporal area; Fu, Fusiform gyrus; Inf T, Inferior temporal gyrus; Inf O, Inferior occipital gyrus.

widespread areas in the occipital lobe and occipitotemporal cortex than Kana words.

PET and functional MRI studies from Western countries also showed similar findings (for review, see Fiez et al. [33] and Price [69,70]): (i) ventral occipital and occipitotemporal cortices, consisting of Areas 18, 19 and 37, were consistently activated by real words, pseudowords (corresponding to Kana nonwords) and letter strings [72,74,95]; (ii) activity of Area 37 was more pronounced in real words relative to letter strings [45], and in real words relative to geometric shapes [95] or to a crosshair [5,18], in contrast to the greater activity of the inferior occipital gyrus (Area 18/19) in letter strings relative to geometric shapes [95] or to a crosshair [5], although in some studies Area 37 was equally activated by pseudowords [32,95], in pronouncing letters relative to saying the same word to false fonts [71], or in viewing letter strings relative to faces [74], and Area 19 was most activated in viewing real words relative to a crosshair [57]; (iii) the middle occipital gyrus (Area 18) was specifically activated by pseudowords and letter strings [5,43,95], and the inner temporal cortex at the superior temporal sulcus (Area 22/21) was also activated in real word reading [13,73], whereas the deep perisylvian cortex under the posterior ramus of the Sylvian fissure (Area 40/22) was activated in real word processing relative to viewing of line drawings [13] or to letter string processing [72] and pseudoword processing relative to letter string processing [72] or to word processing [72]; and (iv) pseudoword processing produced greater activation in the occipital lobe than real word processing [45,72]. Overall, these results suggest that two distinct areas, i.e. the ventral occipitotemporal cortex (Area 37) and inferior occipital gyrus (Area 18/19), are crucial for reading.

Based on the above findings, we hypothesized a weighted dual-route model of reading [80] (Fig. 3) that modifies Iwata's model about Japanese [49]. According to Iwata's model, Kanji are inevitably processed from the primary visual cortex to Wernicke's area by way of the posterior inferior temporal area (semantic pathway or ventral route), whereas Kana are mainly processed from the primary visual cortex to Wernicke's area by way of the angular gyrus (phonological pathway or dorsal route). Iwata's model is along the lines of the dual-route hypothesis for reading in Western countries [19,58,63]. The basic concept is that two parallel pathways, i.e. lexical (semantic) and nonlexical (phonological), are involved in reading, and prior to these pathways, there is a process of visual recognition of words or letters [19,58,63,103].

The modified dual-route model is as follows. The basal occipital (lingual and fusiform gyri, Area 18/19), fusiform and posterior inferior temporal gyri (Area 37) constitute a ventral (orthographic) route for reading and process holistic word recognition that links to lexico-semantics, whereas the lateral occipital/fusiform gyri (Area 18/19), deep perisylvian temporoparietal area (Areas 22/21 and 40/22) and posterior superior temporal gyrus constitute a dorsal (phonological) route for reading and process sequential grapheme-to-phoneme conversion for words. Early visual analysis consisting of line and contour detection and the subsequent letter or character identification are performed in the primary visual cortex and the surrounding visual association cortices in the lingual, fusiform and lateral occipital

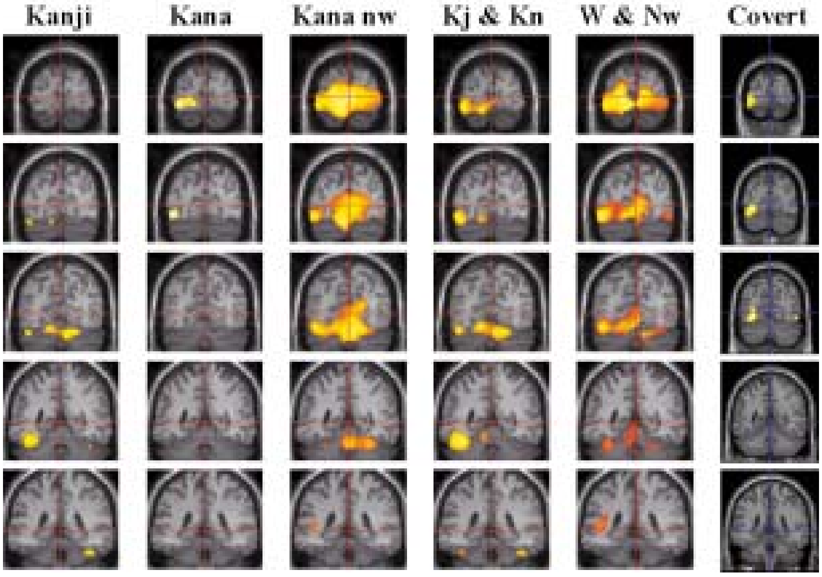

Fig. 2. Activated sites in reading of Kanji words, Kana words, Kana nonwords, Kanji and Kana conjunction, Kana word and nonword conjunction, and covert reading of Kana words [79–81]. Activation is superimposed on the coronal planes in the Statistical Parametric Mapping [37] Version 1996 (reading aloud tasks) or 1999 (covert reading task) template through the calcarine sulcus and lateral occipital gyri (upper two, $y = -90$, $-80$ mm), the angular gyrus (middle, $y = -70$ mm), and the fusiform and inferior temporal gyri (lower two, $y = -50$, $-40$ mm). A vertical line denotes the z-axis ($x = 0$), and a horizontal line denotes the x-axis ($z = 0$) in the atlas of Talairach and Tournoux [96]. Note the activation of the lateral occipital gyri (middle and inferior occipital gyri) in Kana word reading in contrast to the activation of the lateral fusiform/inferior temporal gyri in Kanji word reading. The activation images were slightly different from the previously reported ones [80], because the excluded slices with focal abnormal uptake in the previous Kanji reading task were included in the present analysis. Areas of peak activation, with the x, y, z coordinates in MNI (Montreal Neurological Institute) space and Z-score (x,y,z,Z), are as follows. Kanji words: BA37 (−42,−54,−22,4.37), BA22 (−60,−8,2,4.27), (−60,−20,4,4.13) and (60,−8,2,5.17), BA4 (−44,−14,36,3.69), BA43 (−48,−10,18,4.10). Kana words: BA17 (−20,−100,−8,3.85) and (6,−98,2,3.54), BA18 (−18,−98,8,3.78) and (−32,−94,−8,3.64), BA19 (−44,−80,−12,3.70), BA42/22 (64,−10,8,3.84), BA22 (−62,−22,6,4.13) and (66,−18,6,3.80), BA3 (−50,−14,48,4.70) and (50,−18,40,3.49), BA6 (−58,2,24,4.89), Supplementary motor area (−6,6,54,4.36). Kana nonwords: BA17 (−16,−98,4,6.21), (2,−96,2,5.98) and (16,−72,10,4.45), BA18 (−46,−86,−12,5.35), BA22 (−66,−2,2,5.44) and (72,−20,6,5.34), BA22/21 (−48,−40,10,3.60), (−72,−34,8,4.22) and (−72,−18,−2,3.34), BA4 (−50,−10,48,5.53), BA6 (−54,−6,28,5.32), (62,−12,38,4.53) and (68,−10,30,4.51), BA43 (70,−14, 22,4.16), BA45 (60,26,4,4.24). Kanji and Kana conjunction: BA17 (6,−96,−2,3.66), BA37 (−42,−54,−22,5.24), BA22 (−60,−20,6,5.75) and (62,−8,4,6.10), BA43 (−48,−8,20,6.05), BA4 (−48,−12,46,5.72) and (48,−14,38,4.66), Supplementary motor area (−6,6,54,4.35) and (6,12,54,4.40). Kana word and nonword conjunction: BA17 (−18,−98,4,7.04) and (4,−98,2,6.61), BA18 (−34,−92,−6,5.80) and (46,−84,−10,4.29), BA19 (−44,−82,−14,6.11), BA22 (70,−20,6,5.79), BA22/21 (−48,−42,10,4.44), BA4 (−48,−12,48,7.15) and (−54,−6,24,7.00), BA6 (60,−12,42,5.11), Insula (−28,16,8,4.19), Supplementary motor area (−4,4,54,5.07). BA40/22 was not identified as a separate local maximum in the present data. Kana word covert reading: BA18 (−30,−72,−4,4.68), (−38,−92,−2,4.15), BA19 (−38,−84,−14,4.24), (−8,−82,28,3.22), (48,−68,−6,3.59) and (44,−50,−4,3.18). Abbreviations: nw, nonword; Kj, Kanji; Kn, Kana; W, Word (Kana); Nw, Nonword (Kana).

gyri (the inferior occipital/fusiform gyri [orthographic route] and middle occipital gyrus [phonological route] are continuous in the lateral occipital gyri [Area 18/19] and are also included in the visual analysis system). The phonological lexical information is stored as neural networks around the end of the phonological pathway (posterior superior temporal gyrus), whereas the orthographic lexical information is stored also as neural networks around the end of the orthographic pathway (lateral fusiform and inferior temporal gyri) [5,18, 78]. The posterior superior temporal gyrus and posterior inferior temporal cortex (Area 37) have a reciprocal connection, and facilitate both phonological and orthographic routes in reading (lexical help or lexical capture) [38,66]. Lexico-semantic information is diffusely stored in widespread areas extending from Area 37 to the anterior part of the temporal lobe [27] (semantic storage site in Fig. 3), and Area 37 intermediates between orthography and lexico-semantics.

One main feature of the present model is that the two pathways are not parallel; they change their weight of involvement according to the number of times the word is seen and recognized. That is, Kana nonword and unfamiliar word reading requires both routes. However, as the word is seen and recognized again and again, the whole-word image and meaning are stored around Area

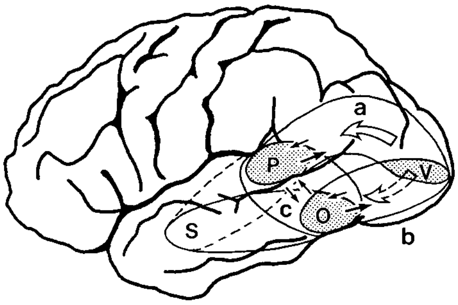

Fig. 3. Diagram of anatomically-based dual-route hypothesis for reading. Early visual analysis and the subsequent letter or character identification are performed in the primary visual cortex and the surrounding visual association cortices in the medial and lateral occipital gyri (V). The phonological pathway (dorsal route; a), which is used for sequential visual recognition of letter or character sequences, proceeds from the primary visual cortex to the posterior superior temporal gyrus (Wernicke's area) via the lateral occipital gyri (middle and inferior occipital gyri) and deep perisylvian temporoparietal cortex. The phonological lexical information (P) is stored around the end of this route. The orthographic pathway (ventral route; b), which is used for holistic visual recognition of words, proceeds from the primary visual cortex to the inferior temporal gyrus via the fusiform gyrus. The orthographic lexical information (O) is stored around the end of this route. The superior temporal gyrus and posterior inferior temporal cortex have a reciprocal connection (c) and facilitate both phonological and orthographic routes in reading (lexical capture or lexical help, illustrated with backward larger-black arrows). The semantic storage site (S), where lexico-semantic information is stored, extends from the posterior inferior temporal cortex to the anterior part of the temporal lobe. During listening, the semantic storage site connects tightly with the posterior superior temporal gyrus (illustrated with dotted lines). The semantic storage site actually includes more extensive areas in the temporal lobe. Reproduced with permission from Sakurai, Shsnkei Kenkyu no shinpo, 2002.

37, thus the ventral route gains dominance in reading real words (Kanji and Kana) [80]. This weighted dual-route hypothesis is mainly supported by the findings that Kana nonwords activated more widespread areas in the ventral and lateral occipital gyri, but not Area 37, than real (Kanji and Kana) words, and that the ventral occipital and occipitotemporal gyri (Areas 18/19 and 37) were predominantly activated by a conjunction involving Kanji and Kana (see Fig. 2 and the summaries (i) and (iv) of our PET studies described above).

In addition, the dorsal posterior occipital gyri (middle and superior occipital gyri) that were activated in Kana word reading relative to Kanji word reading were also activated in Kana reading aloud relative to Kana covert reading, and Kana word covert reading relative to fixation control, although the activation was smaller in covert reading [81]. These facts suggest that dorsal posterior occipital gyri that play the part of earlier phonological processing are more recruited in vocalization tasks.

### 4.3. Anatomical substrates of alexia

In the following section, three types of alexia are described in the light of the present dual-route model. In essence, the two separately activated areas are responsible for alexia: the fusiform/inferior temporal gyri (Area 37) for alexia with agraphia and the fusiform/inferior occipital gyri (Area 18/19) for pure alexia for Kana. Pure alexia for real words results from damage to the fusiform gyrus, medial to the fusiform/inferior temporal gyri. Table 3 summarizes alexias and the supposed lesion sites with references.

#### 4.3.1. Pure alexia: ventromedial type

Damage to the orthographic (ventral) route causes pure alexia with less pronounced letter-by-letter reading [100], and alexia with agraphia for Kanji [49,53, 85,92] or lexical agraphia in Western countries [24] (Table 3).

Pure alexia occurs when the fusiform gyrus under the posterior horn at the occipitotemporal area (Area 37; Fig. 1(a)) is damaged [87,100] and, as a result, the lateral fusiform/inferior temporal gyri are disconnected from the medial fusiform gyrus [87]. As mentioned earlier, visual images of words or orthographic lexical information are probably stored around lateral fusiform/inferior temporal gyri (Area 37) [5,18,78], and widespread areas in the ventral and lateral occipital gyri are concerned with letter or character processing. In this type of pure alexia, therefore, both letter or character identification and whole-word recognition become poor, although still possible. Eventually, impairment is evident chiefly in nonword reading, in which lexical help [38,66] cannot be expected, and many reading errors show visual similarity to the target. Furthermore, letter-by-letter reading is not so pronounced, since the posterior occipital gyri are more specialized to recognize letters or Kana characters and thus the phonological (dorsal) route compensates for any reading deficit.

#### 4.3.2. Alexia with agraphia for Kanji from a posterior inferior temporal cortex lesion

Alexia with agraphia for Kanji [49,53,85], pure agraphia for Kanji [92] or lexical agraphia in Western languages [24], occurs when a lesion is located more laterally in the fusiform and inferior temporal gyri (posterior inferior temporal area or Area 37) in the orthographic route, where visual images of words were suggested to be stored [5,18,78]. In this case, whole-word images of real words (Kanji and Kana) are damaged. Thus, reading depends on the intact phonological pathway. As Kana words and Kana nonwords are also read, although slowly, with the phonological pathway, Kanji word reading is predominantly disturbed. Deficient orthography also affects writing; the patients cannot write, particularly not Kanji words, because of impaired word recall [87]. In this sense, alexia with agraphia for Kanji should be designated "orthographic alexia with agraphia." In addition, since letters or characters are also processed in the ventral occipital gyri, a slight disturbance in letter or character identification coexists with this alexia, which is markedly observed in Kana nonword (or pseudoword) reading (patient 3).

Kinesthetic reading is unsuccessful in alexia with agraphia for Kanji due to a posterior inferior temporal lesion (Table 1), probably because visual images of words per se are disrupted, and thus cannot be recalled [87]. Also, anomia accompanies this type of alexia [53,75,85,92]. However, severer anomia occurs when the lesion extends forward to the anterior third of the temporal lobe or medially to the parahippocampal gyrus [85].

The cooccurrence of anomia and alexia with agraphia for Kanji resembles the symptoms of progressive fluent aphasia [84] or Gogi (word-meaning) aphasia [50] that corresponds to semantic dementia [46] in Western countries. Anomia and surface dyslexia (particular difficulty in irregular word reading) are the prominent features of semantic dementia. An essential difference between progressive Gogi-aphasia or semantic dementia and alexia with agraphia for Kanji or orthographic alexia with agraphia is that semantic dementia derives from loss of semantic memory for words [46,97] (or damage to the semantic storage site in Fig. 3), whereas orthographic alexia with agraphia is an impairment of orthographic lexicon per se. The apparent similarity of symptoms, i.e. Kanji words with atypical reading or European irregular words are read or written more poorly than Kana words or European regular words is probably due to the overlap of the lesion site: the posterior inferior temporal cortex (Area 37).

*4.3.3. Pure alexia for Kana: Posterior type*

Damage to the visual analysis and the earlier stage of the phonological (dorsal) route impairs letter or character identification, thus leading to letter-by-letter reading. As described earlier, letters or characters are processed widely in the ventral and posterior occipital cortices, but the posterior occipital gyri are more specialized to recognize letters or Kana characters [77]. Therefore, letter-by-letter reading is more pronounced in this lesion. Conversely, whole-word reading is possible in the intact orthographic route, thus Kanji characters, which primarily depend on holistic recognition for reading, can be processed by this route. If damage extends to the cuneus or optic radiation, right lower quadrantanopsia may occur.

Pure alexia for Kana parallels pure alexia for letters or syllables, thus for regular words and nonwords, especially for nonwords that are more difficult to read, with preserved reading of real words of which whole-word images are available. Indeed, pure alexia showing a greater impairment for nonwords has been reported in Western countries in patients whose lesion involved the posterior occipital gyri [8,17,30].

Many reported patients with letter-by-letter reading showed the involvement of both the orthographic route and the visual-phonological route. The essential lesion lies in the fusiform gyrus under the posterior horn (Fig. 1(a)) [87,100], but we should note that in many cases the lesion extends posteriorly to involve the lateral occipital gyri (Fig. 1(c)) [30,51,99]. Since recognition of both individual letters or characters and word-forms is disrupted, the patient depends on letter-by-letter reading using the deficient phonological route. Reading is thus slow and laborious. A lesion restricted to the fusiform gyrus extending posteriorly to involve the fusiform/inferior occipital gyri is sufficient to produce letter-by-letter reading, in this case, without visual field defects [44,47,57,101]. If the lesion involves the lingual gyrus or optic radiation, right upper homonymous quadrantanopsia [51,56,76,103] (the present patient 1) or hemianopsia [7,23,34,52,99,103] coexists with alexia.

When the lesion affects the lateral fusiform and inferior temporal gyri, agraphia accompanies alexia (orthographic alexia with agraphia, 4.3.2).

Most of the reported letter-by-letter reading cases were non-classical type pure alexia, in particular preangular alexia without a splenium lesion [42]. The classical spleno-occipital type pure alexia can also cause letter-by-letter reading [1,22,23], if the transcallosal fibers (splenium or forceps major) interrupt visual information going from the right hemisphere to the left fusiform and lateral occipital gyri. With respect to this point, patient 1 did not show pure alexia until his second stroke involving the fusiform gyrus in addition to the first stroke to the lingual gyrus. This finding suggested that the right homologous area did not compensate for the function of the left fusiform gyrus in reading. The inferior portion of the splenium [1] probably conveys visual information that is necessary for word or letter recognition (but is not specific for language) from the right hemisphere to the left fusiform gyrus. Coslett and Saffran [22] stressed the compensatory role of the right hemisphere in reading. However, the left fusiform and inferior temporal gyri seemed spared in all their patients. Therefore, it is possible that reading improved when the visual information on words was transmitted to these gyri through recovered inter- or intrahemispheric fibers.

### *4.4. Similarities and differences among other models of reading*

Several models of reading have been proposed based on classic neurology [40], cognitive neuropsychology [31,35,60,65], connectionism [89] and neuroimaging [70]. Figure 3 illustrates the present model mapped onto brain surface images. This model is anatomically based. Therefore, it is easy to correlate lesions with symptoms. From a neuroanatomical point of view, the flow of information is no longer depicted with a box-and-arrow diagram. There is an overlap of localization between functional units (modules). Therefore, modules with oval-shaped areas overlapping each other were delineated. This model resembles the dual-route model of Ellis and Young [31] or Patterson and Shewell [65] in that subsequent to orthographic analysis, two routes, a direct orthographic lexical route and a sub-lexical route enabling grapheme-phoneme conversion, are postulated. But letter/character identification should not be confined to early visual analysis, as described in 4.1. According to Ellis and Young's model or Patterson and Shewell's model, the orthographic lexical route is subdivided into a lexical (phonological) route, in which a whole-word orthographic lexicon directly accesses a phonological lexicon, and a semantic route, in which the whole-word orthographic lexicon is translated to the phonological lexicon via semantics. In this sense, their model has three routes. This basic architecture is also expressed in Coltheart et al.'s dual-route cascaded model [21].

The present model is different from Ellis and Young's or Patterson and Shewell's mainly in two points. Firstly, in the present model the phonological lexical information (phonological input lexicon) is stored around the end of the phonological route (or sub-lexical route, according to Ellis and Young's or Patterson and Shewell's terminology): sub-lexical grapheme-phoneme conversion is performed in the phonological route, and access to the phonological (input) lexicon results from sequential grapheme-phoneme conversions, whereas in their model grapheme-phoneme conversion has no direct connection with the phonological (input or output) lexicon, Instead, grapheme-phoneme conversion has an access to the phonological output lexicon via the phoneme level (p. 192 of Ellis and Young [31]), but the phonological output lexicon has no backward connection to the semantic system. Thus, their model cannot connect grapheme-phoneme conversion with the semantic system. This means that reading through grapheme-phoneme conversion does not reach semantics. In the present model, converted phonological sequences for a word may go to the inferior supramarginal gyrus and superior temporal gyrus where the phonological lexicon is located, and to this point semantics have an access. If there is no corresponding phonological lexicon in the superior temporal gyrus, the phonological information may be temporarily stored in the supramarginal gyrus [86].

Secondly, the present model assumes direct interaction between the orthographic (input) lexicon and the phonological (input) lexicon and backward connection from the phonological (input) lexicon to the visual analysis, whereas their model has no direct interaction between the two modules or the backward connection. This means that their model does not depict phonological lexical capture, although orthographic lexical capture is represented as a backward arrow from the orthographic (input) lexicon to the visual analysis.

The present model is also different from the dual-route model of McCarthy and Warrington [60] in that two routes do not stem from what they call the whole-word system but from early visual analysis.

In these two models, letter-by-letter reading is explained either by a deficient parallel processing of letters from orthographic analysis to the orthographic input lexicon [64] or by impairment of the whole-word system [103], i.e. a disorder of the orthographic route between visual analysis and the whole-word system (orthographic lexicon). However, these assumptions were suggested to be insufficient to explain sequential letter-by-letter reading [64]. Recently, Friedman and Lott [35] also claimed that letter identification is divided into an explicit serial process and an automatic parallel process, and that letter-by-letter reading is the result of disrupted parallel identification of letters. As the authors noted, this hypothesis alone cannot explain why letter-by-letter reading takes much longer than normal reading. In contrast, the present model explains that letter-by-letter reading occurs using the damaged visual-phonological pathway (letter identification followed by grapheme-phoneme conversion) that is characterized by sequential processing. It is easier for this model to account for why letter-by-letter reading takes much longer.

Namely, both visual analysis (letter identification) and access to the orthographic input lexicon (whole-word reading) are impaired in ventromedial type pure alexia (4.3.1). As a result, the sub-word level grapheme-phoneme conversion process, of which is sequential in nature, compensates for reading impairment. If this process is intact, letter-by-letter reading will not be prominent. In the case of posterior type pure alexia (4.3.3), an area specialized for letter identification is damaged, which directly affects sequential grapheme-phoneme conversion, thus leading to pronounced letter-by-letter reading. In addition, in both types of pure alexia, the orthographic input lexicon itself is affected to some degree, because letter identification and whole-word reading are processed in the areas anatomically close to each other.

One advantage of this model is that it can explain various degrees of letter-by-letter reading [8], depending on the involvement of the visual-phonological route (posterior occipital gyri). Furthermore, in the present model, the orthographic lexicon links to or overlaps with semantics at Area 37 (Fig. 3). Therefore, if this area (fusiform and inferior temporal gyri) is involved, symptoms associated with orthography or semantics may appear. This assumption probably accounts for many observations in pure alexia such as accompanying agraphia [64,98], semantic reading errors [11,16,48,90, 98], categorization of words the patient could not read and semantic priming effects [102], or better recognition of letters in words than in letter strings [14]. It may also explain semantic errors and comprehension of words the patient could not read (understanding without phonology) in alexia with agraphia for Kanji [87]. The author believes that this simple "double-triangle" structure is enough to explain the mechanism of reading.

The connectionist model proposed by Seidenberg and McClelland [89] consists of three functional units that interact with each other: orthographic, phonological and semantic, with additional units, called hidden units, mediating between these units. In their model, however, letter identification and probably whole-word reading are not expressed, and other models are required to simulate letter-by-letter reading or word-length effect [59,67]. In addition, trials to simulate both word-length effect and correct lexical decision have not been successful to date [9].

Price [70] reconciled the classic neurological model [40] with cognitive models [31,35,60,65,89] using functional imaging data, and also proposed an anatomically constrained model. According to this model, Wernicke's area (Area 22/21) sustains nonsemantically mediated speech output, whereas the posterior inferior temporal cortex is involved in lexical, semantically mediated speech output. Access to semantics is made in the angular gyrus and anterior inferior and middle temporal gyri. This model differs from the present model mainly in two points: (i) the angular gyrus is engaged in semantics; and (ii) the site for the function of Wernicke's area is the upper bank of the posterior superior temporal sulcus (Area 22/21). The author's view is that: (i) the angular gyrus is less involved in reading; crucial areas for reading in this vicinity are the middle occipital gyrus (posterior to the angular gyrus) and the deep perisylvian temporoparietal junction (Areas 22/21 and 40/22; anterior to the angular gyrus) [80]. Moreover, an essential region for lexico-semantics is the anterior part of Area 37 (MNI coordinates: $x = -52$, $y = -44$, $z = -18$), 10 mm anterior to the maximal activation locus of Area 37 for reading [79]; and (ii) the inner temporal cortex (Area 22/21) is not Wernicke's area itself, but posterior to Wernicke's area which is by definition located in the posterior half or third of the superior temporal gyrus (Area 22). The supposed Wernicke's area (peak activity, $x = -60$, $y = -20$, $z = 6$ in a conjunction involving Kanji and Kana) and the inner temporal cortex at the superior temporal sulcus were simultaneously activated in our PET reading studies [80]. It is suggested that the inner temporal cortex (Area 22/21) provides access to the phonological lexicon that is distributed around the posterior superior temporal gyrus.

## 5. Concluding remarks

Visual perception was examined in detail only to patient 2. This patient had a minimal deficit in discriminating between two shapes of a similar size, as described. Based on the fact that the impairment was limited to minute visual shape discrimination, we thought that letter/character forms themselves were disrupted [77]. But it was also possible that this impaired early visual analysis affected letter/character identification more or less. Further study is required to elucidate the role of early visual analysis in reading.

## Acknowledgements

I am grateful to Dr. Toru Mannen, Mitsui Memorial Hospital, for his support and encouragement.
